# CO_2_ induced phase transitions in diamine-appended metal–organic frameworks†
†Electronic supplementary information (ESI) available: Data for images and coordinates. See DOI: 10.1039/c5sc01828e



**DOI:** 10.1039/c5sc01828e

**Published:** 2015-06-17

**Authors:** Bess Vlaisavljevich, Samuel O. Odoh, Sondre K. Schnell, Allison L. Dzubak, Kyuho Lee, Nora Planas, Jeffrey B. Neaton, Laura Gagliardi, Berend Smit

**Affiliations:** a Department of Chemical and Biomolecular Engineering , University of California , 201 Gilman Hall , Berkeley , California 94720 , USA . Email: berend.smit@epfl.ch; b Department of Chemistry , Chemical Theory Center and Supercomputing Institute , University of Minnesota , Minneapolis , Minnesota 55455-0431 , USA . Email: gagliard@umn.edu; c Department of Chemistry , Norwegian University of Science and Technology , Høgskoleringen 5 , 7491 Trondheim , Norway; d Molecular Foundry , Lawrence Berkeley National Laboratory , One Cyclotron Road , Berkeley , California 94720 , USA; e Department of Physics , University of California , Berkeley , USA; f Kavli Energy NanoSciences Institute at Berkeley , Berkeley , CA , USA; g Institut des Sciences et Ingénierie Chimiques , Valais , Ecole Polytechnique Fédérale de Lausanne (EPFL) , Rue de l'Industrie 17 , CH-1950 Sion , Switzerland

## Abstract

Using a combination of density functional theory and lattice models, we study the effect of CO_2_ adsorption in an amine functionalized metal–organic framework. These materials exhibit a step in the adsorption isotherm indicative of a phase change. The pressure at which this step occurs is not only temperature dependent but is also metal center dependent. Likewise, the heats of adsorption vary depending on the metal center. Herein we demonstrate *via* quantum chemical calculations that the amines should not be considered firmly anchored to the framework and we explore the mechanism for CO_2_ adsorption. An ammonium carbamate species is formed *via* the insertion of CO_2_ into the M–N_amine_ bonds. Furthermore, we translate the quantum chemical results into isotherms using a coarse grained Monte Carlo simulation technique and show that this adsorption mechanism can explain the characteristic step observed in the experimental isotherm while a previously proposed mechanism cannot. Furthermore, metal analogues have been explored and the CO_2_ binding energies show a strong metal dependence corresponding to the M–N_amine_ bond strength. We show that this difference can be exploited to tune the pressure at which the step in the isotherm occurs. Additionally, the mmen–Ni_2_(dobpdc) framework shows Langmuir like behavior, and our simulations show how this can be explained by competitive adsorption between the new model and a previously proposed model.

## Abstract 2

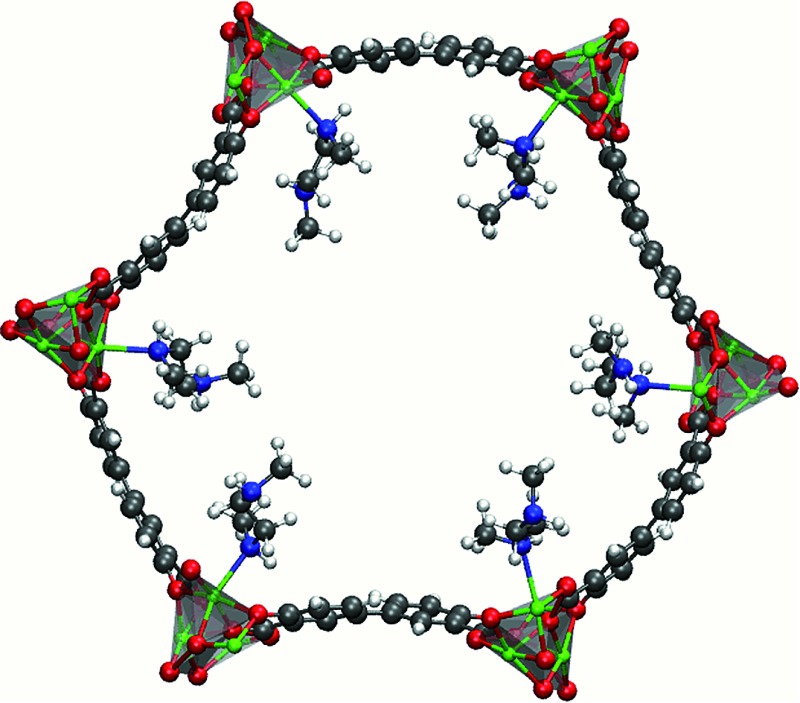
Using a combination of density functional theory and lattice models, we study the effect of CO_2_ adsorption in an amine functionalized metal–organic framework.

## Introduction

As carbon capture and sequestration is the only viable technology to reduce CO_2_ emissions associated with the use of fossil fuels, a great deal of research has been devoted to the development and optimization of emergent technologies to capture CO_2_ from flue gases.^1,2^ In fact, the natural gas industry has been utilizing the well-known ability of monoethanolamine (MEA) to capture CO_2_ since 1930.^3^ The capability now exists to use this technology to capture CO_2_ from flue gas and it is the only technology currently advanced enough for use in power plants. However, while amines are highly effective at separating CO_2_ from other flue gases, the regeneration of amine solutions requires boiling, which is an energy intensive step. Solid sorbents, like metal–organic frameworks, are highly promising as these materials require less energy to be regenerated.^4–9^ While much work has focused on designing new porous materials for CO_2_ capture, another approach is to functionalize a nanoporous material with amines with the aim of combining the selectivity of the amine while maintaining the low regeneration energy requirement of the porous framework.^10,11^

**Fig. 1:**
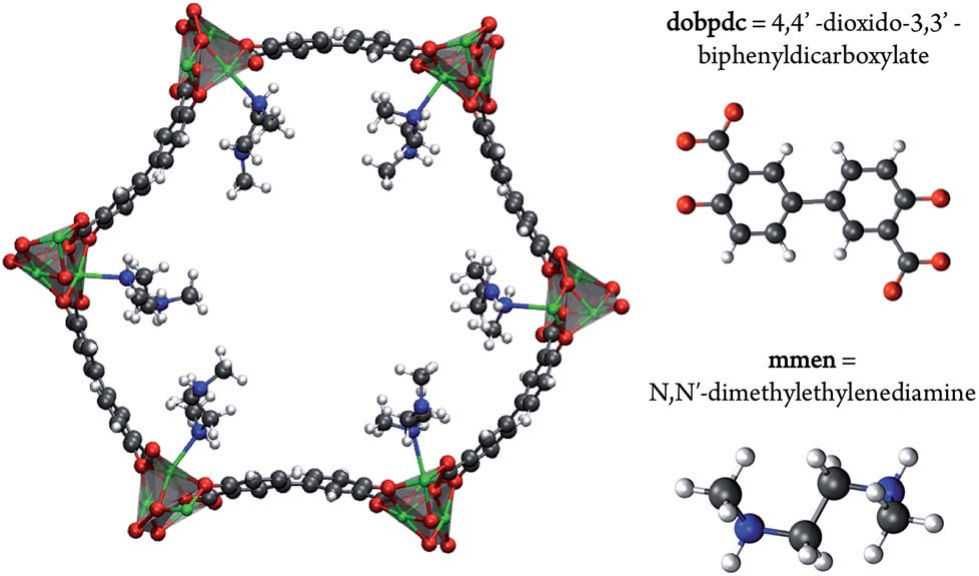
The hexagonal channel of mmen–Mg_2_(dobpdc). The *ab*-plane is in the plane of the paper and the *c*-axis is perpendicular to the hexagonal channel. The amine ligands are coordinated to adjacent metal centers. Mg is shown in green (silver polyhedra), O in red, N in blue, C in grey, and H in white.

To this end, the use of amine-grafted MOFs for CO_2_ capture to design advanced solid adsorbents represents one of the most exciting uses of this class of compounds. The mmen–M_2_(dobpdc) framework (4,4′-dioxidobiphenyl-3,3′-dicarboxylate; mmen = *N*,*N*′-dimethylethylenediamine, M = Mg, Mn, Fe, Co, Ni, Zn) is of particular interest due to its stability in water and unique CO_2_ adsorption mechanism (see Fig. 1). For some metals these materials exhibit a step in the adsorption isotherm indicative of a phase change.^11^ From a practical point of view, such a step in the adsorption isotherm is ideal as a small change in (partial) pressure induces a large change in the amount of gas adsorbed. However, to take advantage of such a step one needs to be able to select a material that has the step at exactly the right conditions for a given separation. A prerequisite for the rational design of such a material is a better understanding of the molecular mechanism for CO_2_ adsorption in mmen–M_2_(dobpdc). In this context an important question is to have a quantitative understanding why the Mg, Mn, Fe, Co, and Zn versions of the mmen–M_2_(dobpdc) have a step in the isotherm, while Ni does not. Recently, Lee *et al.* have synthesized dmen–Mg_2_(dobpdc) (dmen = *N*,*N*-dimethylethylenediamine) and also observe this characteristic step in the isotherm.^12^ In this work, we report a combined quantum chemical and classical simulation study aimed at elucidating the manner in which CO_2_ binds to the mmen–M_2_(dobpdc) framework, but the underlying concepts are extendable to the other amine appended MOFs in the amine appended M_2_(dobpdc) family. Some initial results of this work have been published as part of an extensive experimental study and we will note explicitly in the following which results were previously published and which are included herein for the first time.^11^

## Results and discussion

**Fig. 2:**
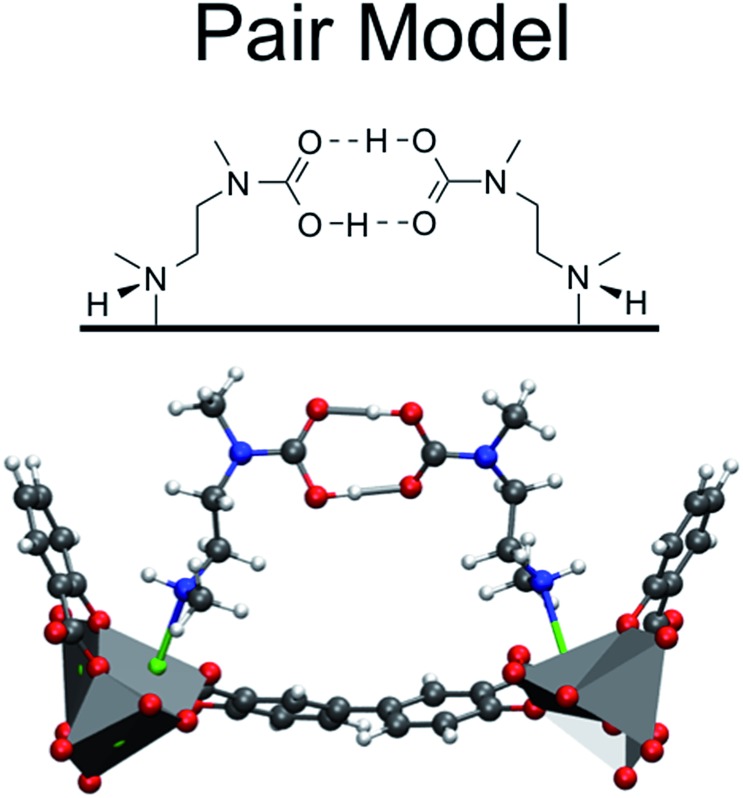
The pair model proposed in the work of Planas *et al.* ^13^ DFT calculations were performed on periodic unit cells.

The first step in understanding the unique behavior of mmen–M_2_(dobpdc) is to determine the manner in which CO_2_ binds to the amines in the framework. Given the open metal site in the MOF, it is reasonable to assume that one of the amine is bound to the metal site and the other amine will adsorb CO_2_. With this assumption, the first experiments performed by McDonald *et al.* for the Mg framework showed that CO_2_ adsorption occurs with 1 : 1 (CO_2_ : amine) stoichiometry, in contrast to the 1 : 2 ratio observed for aqueous amine solutions, and has a heat of adsorption of –71 kJ mol^–1^.^10^ Given that one of the amine groups is assumed to be strongly bound to the open metal site and that it is not involved in the interaction with CO_2_, this 1 : 1 ratio is surprising. To explain this observation, Planas *et al.* ^13^ performed a density functional theory (DFT) study and proposed a possible adsorption mechanism. In Planas *et al.*'s work, the amine was assumed to be grafted to the open metal center with only its terminal end free to capture CO_2_, and a binding configuration involving a carbamic acid pair was reported. We refer to this structure as the *pair model* (see Fig. 2). CO_2_ capture could occur in the *ab*-plane (across the organic linker) or by forming the same pair between neighboring amines along the *c*-axis (see Fig. 1).^13^ Pair formation across the *ab*-plane was in good agreement with the experimental data available at the time (the heat of adsorption and the 1 : 1 CO_2_ : amine ratio).

Subsequent experimental data however have cast doubt on whether only one amine group is participating in the binding of CO_2_. First, the experiment was repeated for the transition metal analogues (mmen–M_2_(dobpdc) where M = Mg, Mn, Fe, Co, Ni, and Zn) and the heat of adsorption was shown to be metal dependent. This was inconsistent with CO_2_ binding only to the terminal end of the amine. Additionally, the CO_2_ adsorption isotherm has a characteristic step for all of the metals with the exception of nickel, and this step is indicative of a phase change mechanism in which the amines switch from an initially disordered state to some ordered structure. The pressure at which this change occurred was metal dependent and could not be fully explained by the proposed pair mechanism. Likewise, ^15^N NMR data suggest that CO_2_ adsorption affects how the amine coordinates to the MOF. At temperatures relevant for CO_2_ storage, only one peak is observed in the ^15^N NMR spectra despite the fact that one nitrogen atom is coordinating to the metal center and the other is not. The presence of only a single peak indicates that the amine can alter its coordination at a time scale faster than the NMR can resolve.^11^

These new experimental data suggest that the amine group bound to the metal site must participate in the binding of CO_2_. First, to quantify the different binding modes and the effect of changing the metal, we have carried out a thorough quantum chemical study to identify the binding mechanism of CO_2_ in this framework. Ultimately, we aim to go beyond a static picture of the binding mode by performing molecular simulations. Can we demonstrate that the product identified by our calculations and the work of McDonald *et al.* ^11^ yield the transition observed in the isotherm? Can we predict a change in the adsorption mechanism by tuning the energetics?

### CO_2_ binding in mmen–Mg_2_(dobpdc)

**Fig. 3:**
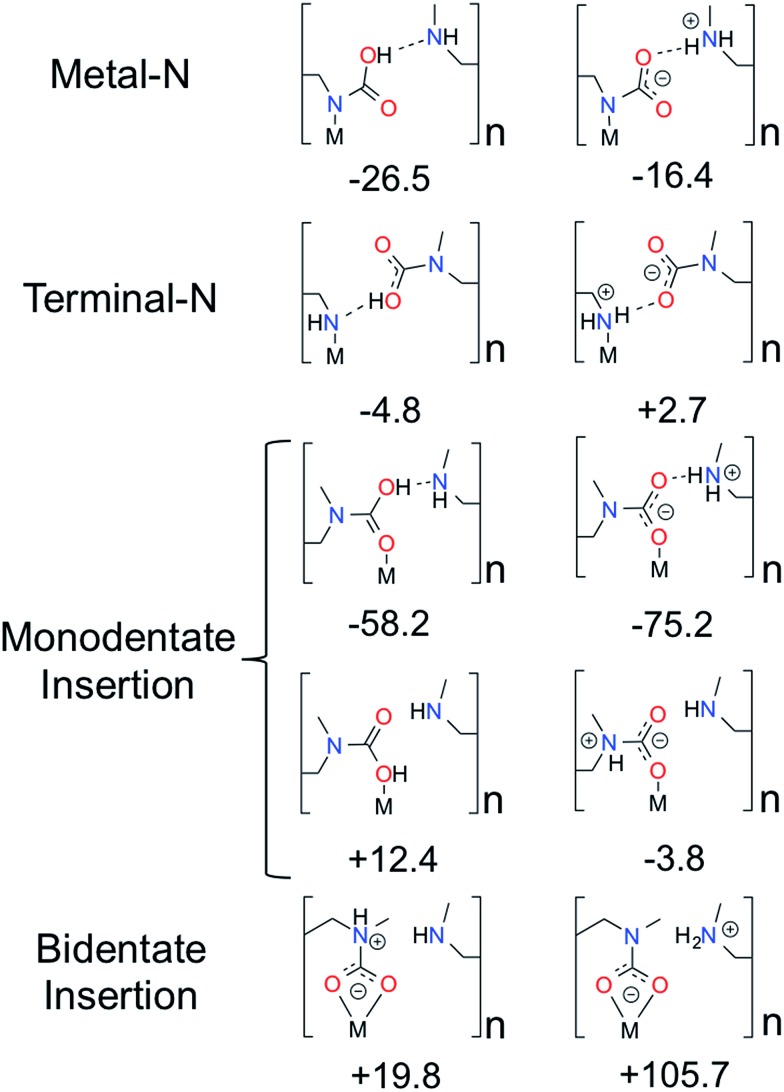
Possible ways for CO_2_ binding in mmen–Mg_2_(dobpdc) to form a chain explored in this work. CO_2_ binding energies are reported in kJ mol^–1^ and were computed with the PBE functional^15^ on the unit cell using periodic boundary conditions. The structures in the first column differ from the second column by a proton transfer.

An alternative to the pair model that also explains the 1 : 1 CO_2_ : amine ratio is a model in which CO_2_ binds at the metal site with two amine groups. In this model, CO_2_ coordinates to the metal bound nitrogen but has favorable interactions with the neighboring amine down the *c*-axis forming a highly ordered chain and is therefore referred to as the *chain model*. However, there is more than one way to form an ordered “chain” structure, and one can show that there are ten such binding modes of CO_2_ that maintain the 1 : 1 CO_2_ : amine ratio (Fig. 3). The ten structures consist of all possible ways that CO_2_ can bind to either end of the amine, eliminating structures that are obviously high in energy (*e.g.* a positively charged nitrogen atom coordinating to a positively charged metal). The rationale for choosing the ten structures is as follows. Four categories of CO_2_ binding modes in mmen–Mg_2_(dobpdc) have been identified. CO_2_ can (1) bind to the nitrogen atom coordinated to metal center (Metal–N), (2) bind to the terminal nitrogen group (Terminal–N), or (3) and (4) insert into the metal–nitrogen bond. For the latter, CO_2_ insertion can result in either (3) a monodentate product (Monodentate Insertion) or (4) a bidentate product (Bidentate Insertion). One would expect the binding of CO_2_ at the Metal–N and Terminal–N sites to be similar to the well-known reactions that occur in solution between CO_2_ and amines like MEA. MEA solutions have been used commercially for CO_2_ separations for many decades and the mechanism for their reaction with CO_2_ includes the formation of a zwitterion, an ammonium carbamate intermediate;^14^ therefore, both charged and neutral groups are considered as possible products in the Metal–N and Terminal–N groups. Furthermore, for the Monodentate Insertion group, four structures can be formed, two carbamates and two carbamic acids (Fig. 3). The two acid structures differ from one another by which oxygen is coordinated to the metal, the OH or the oxo. For both Monodentate Insertion and Bidentate Insertion, two carbamates can form by protonating either the nitrogen closest to the metal or the terminal nitrogen. Only carbamates are considered for the Monodentate Insertion group since the analogous carbamic acid is unlikely to form. For all of the structures in Fig. 3, binding energies were computed at the periodic PBE/DFT level of theory.^15^

**Fig. 4:**
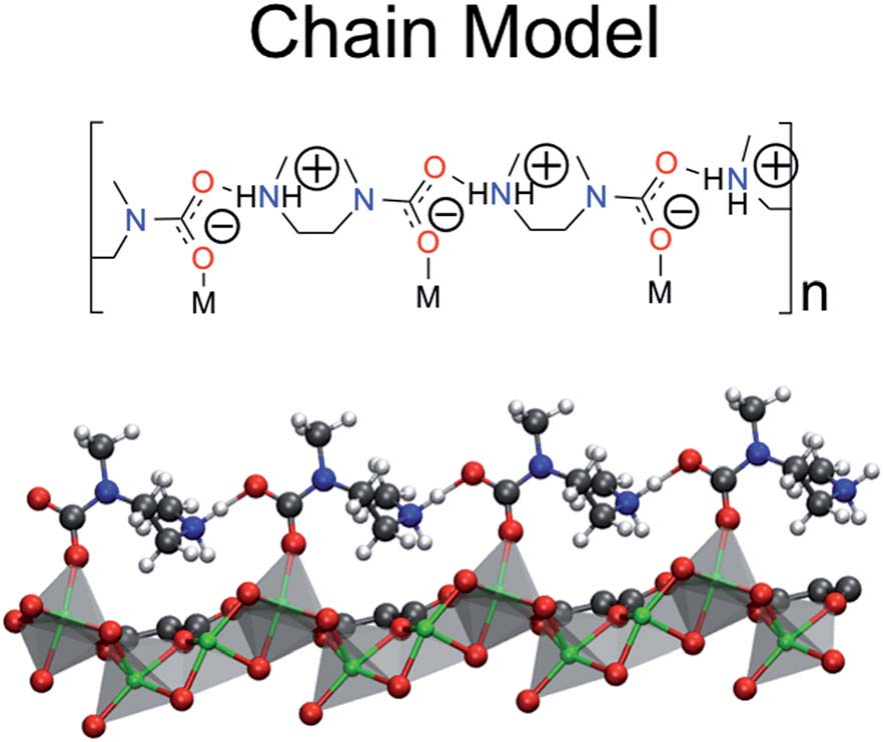
The chain model is the proposed product of the ten structures considered in Fig. 3. The ball and stick model is truncated for clarity only.

The Terminal–N and Bidentate Insertion groups can be immediately excluded as possible products as their formation is energetically unfavorable. The bidentate coordination modes are particularly high in energy at +105.7 and +19.8 kJ mol^–1^, respectively. Likewise, the neutral Terminal–N configuration has a binding energy of –4.8 kJ mol^–1^ while the charged species is unfavorable at +2.7 kJ mol^–1^. Additionally, two of the Monodentate Insertion products can be ruled out since protonating the oxygen atom coordinating to the metal center or positioning the ammonium group close to the carbamate is unfavorable, resulting in energies of +12.4 and –3.8 kJ mol^–1^, respectively. On the other hand, favorable binding is observed for the remaining Monodentate Insertion products and for the Metal–N group. The Metal–N products have binging energies of –16.4 and –26.5 kJ mol^–1^ for the charged and neutral species, respectively. Recall that the experimentally observed heat of adsorption is –71 kJ mol^–1^.^10^ While the formation of a carbamic acid group is favorable at the metal-bound nitrogen, the Mg–N_amine_ bond distances are quite long leading to weaker binding energies. However, if the Mg–N_amine_ bond breaks upon CO_2_ adsorption and the CO_2_ oxygen coordinates to the metal, binding energies of –58.2 and –75.2 kJ mol^–1^ are observed for the neutral and charged products, respectively. The importance of a minimum energy structure that forms a chain-like interaction down the *c*-axis will be addressed when the Monte Carlo results are presented; however, we emphasize again that several of the structures from Fig. 3 meet this criteria. However, in the following the *chain model* will be used only to refer to the lowest energy structure with the binding energy of –75.2 kJ mol^–1^ (shown in Fig. 4 in more detail), where the amines cooperatively bind CO_2_ molecules to form ammonium carbamate chains down the hexagonal channels in the MOF.^11^ Based on these energetics, we propose that CO_2_ first physisorbs to the amine and then forms a carbamic acid at the metal-bound nitrogen, before ultimately inserting in the metal–nitrogen bond.

Additionally, the chain model has been compared to the pair model proposed by Planas *et al.* (Fig. 2 and 4).^13^ In the previous study, the mechanism for pair formation was explored. While a more detailed discussion can be found in ref. 13, CO_2_ first physisorbs at the terminal end of two amines. The carbon of CO_2_ interacts with the lone pair of the nitrogen on one amine while the oxygen forms a hydrogen bond with the NH group on the other amine. The subsequent step is the formation of a carbamic acid group (–COOH) and this step has a barrier of +37.59 kJ mol^–1^. The resulting product is lower in energy by –29.76 kJ mol^–1^ with respect to the separated reactants (or 47.77 kJ mol^–1^ lower in energy than the transition state barrier). The second CO_2_ forms favorable interactions with the –COOH hydrogen and the nitrogen of the other amine. The barrier to form the second –COOH group is similar to the first, +40.38 kJ mol^–1^. The final step proposed in ref. 13 is a rearrangement. The initial reactions were between two amines aligned down the *c*-axis; however, the final product (denoted the pair model herein) includes the “pairing” of two carbamaic acid groups across the *ab*-plane. In the previous work, this was determined to yield a binding energy of –138.25 kcal mol^–1^ with respect to reactants, or –69.13 kcal mol^–1^ per CO_2_. The reader should note that the values are electronic energies based on the cluster model from previous work. After this work was published, two new experimental results were the first to call this mechanism (and resulting product) into question. First, the heat of adsorption varied with the metal, and second ^15^N NMR data suggested that the two N atoms on the amine were equivalent. Both of these would be unlikely if CO_2_ reacted with the terminal end of the amine.^11^ Neither of these results would not be expected if CO_2_ reacts with the terminal end of the amine.

Given that the work of Planas *et al.* employed a cluster model, in the present study we fully optimized products and reactants with periodic boundary conditions. The formation of the dicarbamic acid product (the pair model) is exothermic by only –42.9 (or –45.8) kJ mol^–1^ per CO_2_ with the PBE (or M06L) functionals, respectively. First of all, an important difference between these calculations is that the framework atoms were kept rigid in the cluster model and secondly the cluster model most likely omits steric interactions between adjacent carbonated amines along the *c*-axis. Both of these effects could contribute to the differences between these calculations. As such, the calculated energies obtained for the pair model with the PBE or MO6L functionals and periodic boundary conditions are not in agreement with the experimental enthalpy of –71 kJ mol^–1^.^10^ It should also be noted that the lowest energy structure obtained with these functionals is a zwitterionic ammonium carbamate species (Fig. 4). Charge separated species were attempted by Planas *et al.* but were not stable at the cluster level.^13^

Furthermore, infrared spectra have been measured for mmen–Mg_2_(dobpdc) both before and after CO_2_ adsorption at a variety of temperatures. The predominant features supporting the formation of a carbamate group include two diagnostic bands at 1334 cm^–1^ and 658 cm^–1^ that can be assigned to *ν*(C–N) and [β(OCO) + β(NCO)] modes.^11^ While the region between 1000 and 1600 cm^–1^ cannot be used to distinguish between the pair and chain models as both contain carbamate groups, the calculated carbamate C=O stretching mode, the amine methylene C–H stretches, and the carbamate C–N_amine_ stretching modes occur at 1565.0 ± 100, 1440.6 ± 100, and 1265.9 ± 100 cm^–1^, respectively, in the chain model confirming the assignment of the 1690 and 1334 cm^–1^ peaks as the C=O and C–N_amine_ vibrations of the carbamate.^11^ Additionally, the most intense experimental peak due to product formation occurs at about 2250 cm^–1^.^11^ The pair model has an intense carbamic acid O–H stretch at 2582.7 ± 100 cm^–1^, while the analogous peak in the chain model occurs at 2158.2 ± 100 cm^–1^. As was the case for the binding energies, the chain model is in better agreement with experiment.

### Metal center dependence

Working with the M_2_(dobpdc) family is particularly convenient since this MOF topology can be readily synthesized with many first row transition metal atoms. In M_2_(dobpdc), trends in the lattice constant as a function of changing the metal follow the same trend observed in the analogous family of MOFs, M_2_(dobdc) where dobdc is the 2,5-benzenedicarboxlyate linker.^11^ The M_2_(dobdc) and M_2_(dobpdc) frameworks differ only by the length of the linker. In both cases, lattice constants are longer for metals with a larger ionic radius (Mn > Fe > Co > Zn > Mg > Ni). For example, the difference in the length of the unit cell is 0.29 Å between Mn and Ni in M_2_(dobpdc) compared to 0.33 Å in M_2_(dobdc). On the other hand, when the six amine groups are added to the unit cell, the lattice constants are on average 0.36 Å shorter in the *ab*-plane than in M_2_(dobpdc) but 0.16 longer in the *c*-direction for the same metal.

Currently, CO_2_ adsorption in mmen–M_2_(dobpdc) has been performed for M = Mg, Mn, Fe, Co, Ni, and Zn and the adsorption behavior shows a strong metal center dependence. However after CO_2_ adsorption, powder X-ray diffraction data is only available for the Mn analog.^11^ Most notably, the XRD results show an elongation of the M–N_amine_ bonds from ∼2.44 Å in the bare MOF to ∼4.43 Å after CO_2_ adsorption and the structure is consistent with our chain model. The binding geometries and energies for the chain model calculated within DFT using the PBE and M06-L functionals were discussed in our previous combined experimental and theoretical study.^11^ Our DFT calculations for the pair model were not presented in previous work but were used to justify the choice of interaction energies for the molecular simulations.

**Fig. 5:**
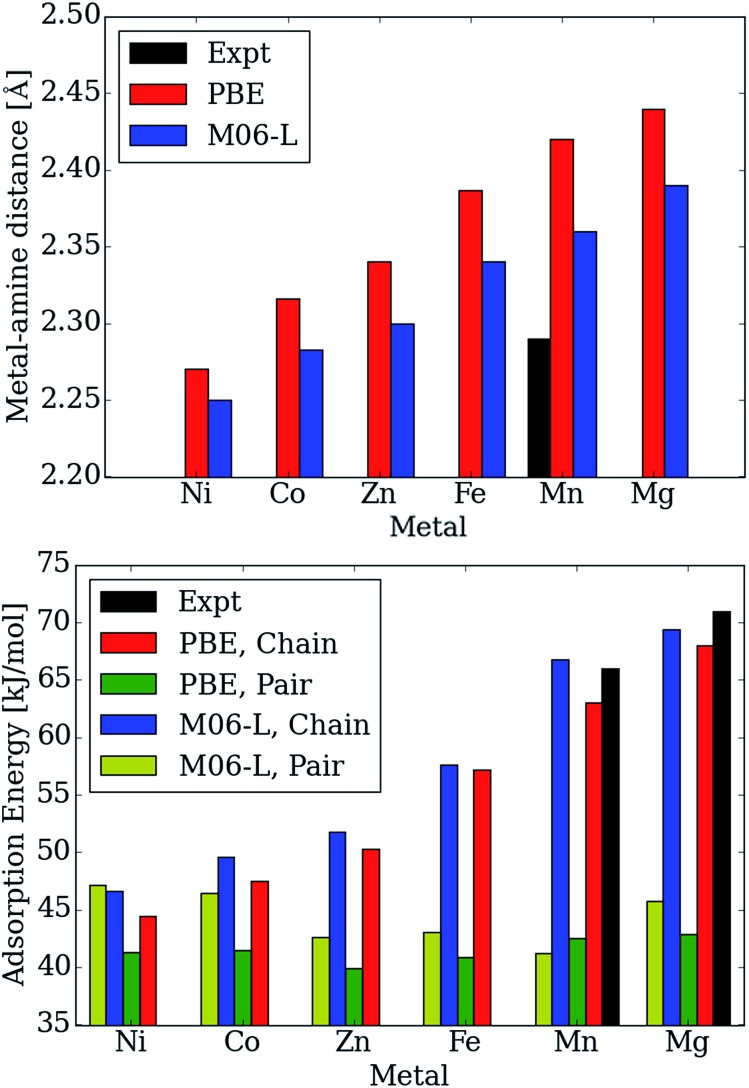
Calculated and experimental adsorption energies in kJ mol^–1^ and metal–N_amine_ distances in Å as a function of metal type.

Moreover, the CO_2_ binding energies and metal–nitrogen bond distances correlate with one another and periodic trends are consistent with well-established behavior for first row transition metals atoms (see Fig. 5). According to the Irving–Williams series for relative stabilities of high-spin divalent metal ion complexes, a shorter bond indicates a more stable species. Note that the M–N_amine_ distance decreases from Mg to Ni (see Fig. 5 (top)). When the M–N_amine_ bond strengthens, CO_2_ insertion becomes less favorable since the reactant is stabilized with respect to the product. This trend further correlates with the number of unpaired 3d electrons at each transition metal center. Metal sites with higher spin densities have larger Pauli repulsion interactions between the singly occupied 3d orbitals and the occupied σ-donor orbital of the amine nitrogen atoms. Since the mechanism for CO_2_ insertion requires the cleavage of the M–N_amine_ bonds, we expect (and observe) frameworks with shorter M–N_amine_ bonds to have less favorable energetics for the CO_2_ insertion step. In Fig. 5 (bottom), we compare the adsorption energy for both the chain and pair model using the PBE and M06-L functionals. For Ni, the M06-L energies favor the formation of pairs over chains. On the other hand for Co, the formation of chains is favored, but the difference is not as significant as it is for the other metals atoms considered here. The PBE functional favors the formation of chains for all of metal atoms considered here.

Additionally, the calculated Mg–N_amine_ bond lengths in the pair model remain virtually unchanged (contraction by only 0.00–0.08 Å) from the reactant to the pair model product. Furthermore, the adsorption energies and Mg–N_amine_ bond distance are independent of the metal for the pair model in mmen–M_2_(dobpdc) contrary to experiment (Fig. 5).

### Origin of the phase-change adsorption isotherm

Our DFT calculations show that the chain model can account for some of the structural and energetic experimental observations. The next step is to show that this model also results in the characteristic step in the adsorption isotherm observed for mmen–M_2_(dobpdc) for M = Mg, Mn, Fe, Zn, and Co.

**Fig. 6:**
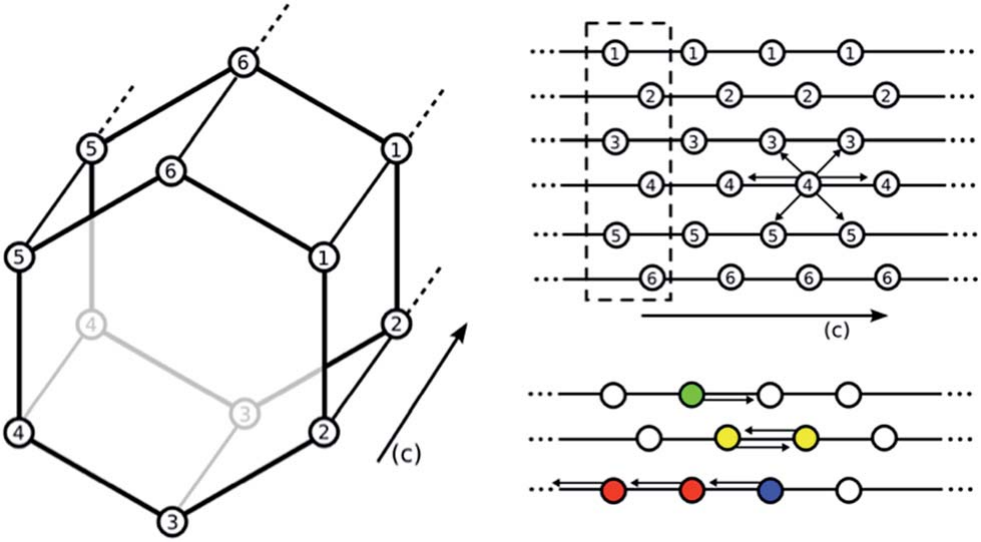
Depiction of the hexagonal channel in M_2_(dobpdc). Lattice points correspond to the position of the metal centers. Empty circles indicate an amine without a CO_2_ bound. Each amine can interact with six neighboring amines (either down the *c*-axis or across the *ab*-plane). All centers with the same number correspond to metal centers ordered along the same *c*-axis. For example, an interaction between 4–4 is in the *c*-direction while an interaction between 3–4 is in the *ab* plane. A white circle indicates an amine without CO_2_ bound. Colored amines all have CO_2_ bound. Green indicates CO_2_ bound to a single amine, yellow indicates the formation of a pair, red is an amine with CO_2_ bound in a chain, and blue is an amine with CO_2_ bound but at the end point of a chain. Interaction energies are based on DFT energetics. The dotted box indicates one unit cell.

To investigate how the two proposed CO_2_–amine complexes influence the shape of the isotherms, we have extended the approach of Sillar *et al.* ^16^ to include the energetics of the complexes proposed in the previous section (see Fig. 2 and 4) in a lattice model. In our lattice model (see Fig. 6) we assume that the amines are grafted on the metal sites and the amine–metal site can adsorb a CO_2_ molecule. The energetics of this CO_2_ adsorption depends on the complexes we assume can form. The amine can point towards a neighboring metal site across the linker (*ab*-plane, 4 orientations) or along the *c*-axis (2 orientations). In the pair model, we assume that two CO_2_ molecules can only adsorb if the two amines on neighboring adsorption sites point towards each other. In the chain model, we assume that CO_2_ is optimally adsorbed in a chain-like configuration (the chain model) giving the full energy contribution of 100%; a CO_2_ at the end of a chain is less favorable (80% of the full-chain energy), as is an isolated adsorbed CO_2_ molecule (24% of the full-chain energy). The energy for a fully formed chain is taken to be equal to the binding energies for the different metals shown in Fig. 5 using the M06-L functional. We used grand-canonical Monte Carlo simulations to compute the isotherms.^17^ The chemical potentials of our lattice model were scaled to reproduce the chemical potential of CO_2_ in the gas phase.

**Fig. 7:**
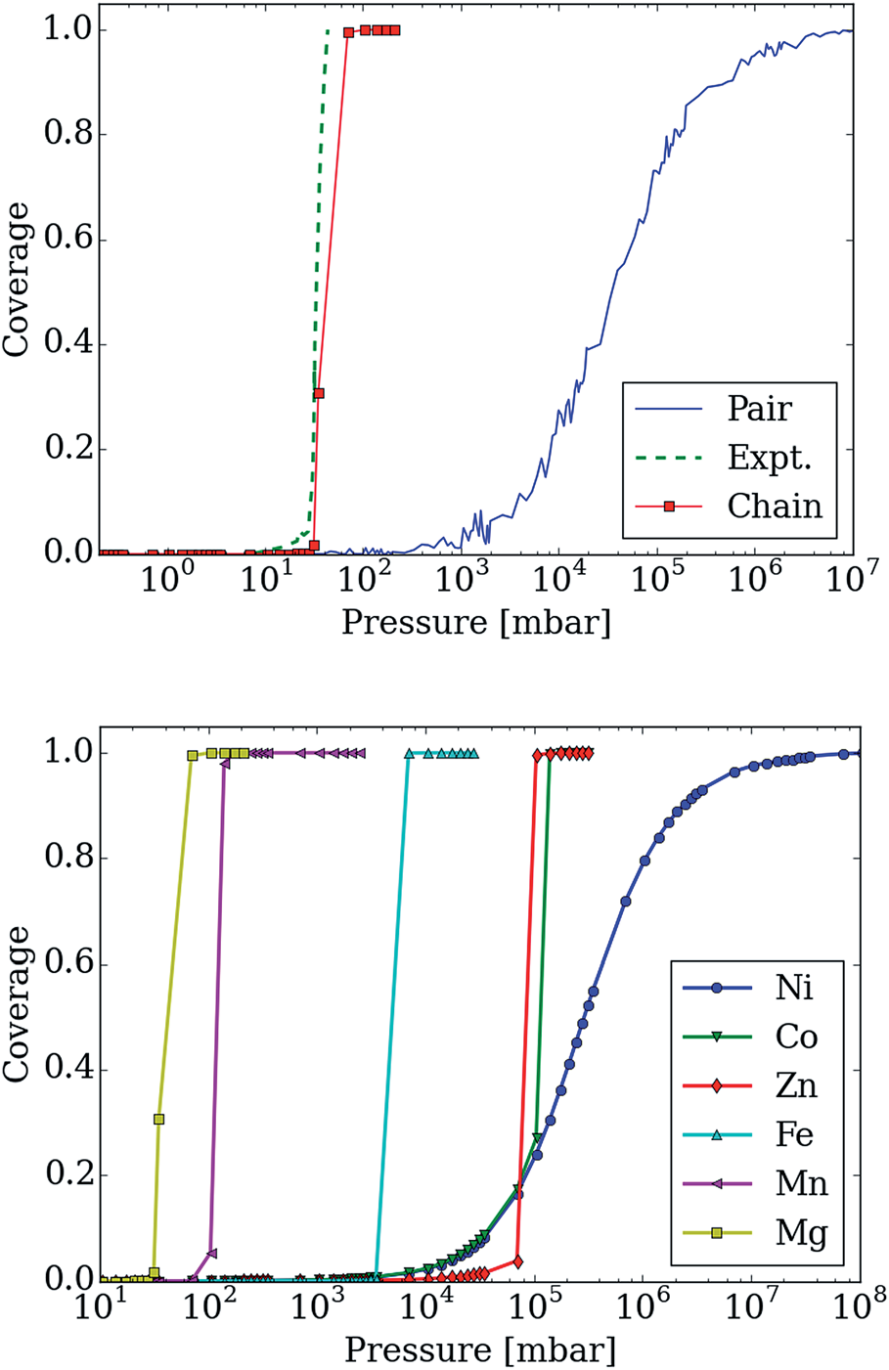
Adsorption isotherms for adsorption of CO_2_ in mmen–M_2_(dobpdc) for M = Mg, Co, Fe, Mn, Ni, and Zn using the lattice-model with interaction energies based on those computed with the M06-L functional. (top) The adsorption isotherm based on the chain model is compared with the experimental isotherm as well as with the pair-model for mmen–Mg_2_(dobpdc). The step in the adsorption isotherm cannot be explained from the pair-model but can be with the chain model. (bottom) Metal dependence in the chain model. The order of the step in the adsorption isotherm corresponds to the order found experimentally from McDonald *et al.* ^11^ Nickel shows a Langmuir like adsorption isotherm, while Co initially shows Langmuir like behavior, before the ordering becomes important enough. The step for Co is found at approximately the same pressure as for Zn; this is in agreement with the chain energy found for Zn and Co at the DFT level of theory that is approximately the same value. However, Co has a higher energy for forming pairs and this will be more important initially.

In Fig. 7 (top), we compare the experimental adsorption isotherms of CO_2_ in mmen–Mg_2_(dobpdc) at 298 K with the simulated adsorption isotherm of CO_2_ for all of the metals, as well as for the pair model. If only the pair model is included in the simulations, conventional Langmuir behavior is observed, while simulations employing the chain model result in the distinctive step observed experimentally. It should be noted that our previous study included the comparison of the pair and chain models for the mmen–Mg_2_(dobpdc) framework.^11^

However, it is quite possible that pair and chain formation are in competition; therefore, the energies to form the chain and pair models were based on the computed DFT values and both possibilities were allowed. In mmen–Mg_2_(dobpdc), chain formation is more favorable than pair formation resulting in behavior qualitatively different from a Langmuir isotherm due to the formation of chains (see Fig. 7) that induce a collective behavior in which the lowest energy configuration is only found if all of the amines align. On the other hand, in the pair model one only needs to form pairs to have the optimal adsorption and pairing does not result in sufficient ordering to yield a step in the isotherm.

Moreover, changing the metal influences the location of the step of the isotherm: the smaller the binding energy the higher the pressure of this step. The experimental results and our calculated results in Fig. 7 (bottom) demonstrate that by carefully selecting the metal site one can tune the pressure at which the transition in the isotherm occurs and such tunability can be of great importance for practical applications. In the coarse-grained model, the energy contribution from the terminal end of a chain is one of the input-parameters that can be specifically tuned. This is the energy an amine at the end of chain contributes to the total energy of the system. For the chain model of mmen–M_2_(dobpdc) shown in Fig. 6, we used an end-point energy that was 80% of the full-chain energy. The reader should note that the results for Mg, Mn, Fe, and Co included in Fig. 7 (bottom) were presented in our combined experimental/theoretical study,^11^ but the results for Ni and Zn were not.

Additionally, the experimental isotherm for nickel is of particular interest since it does not contain the characteristic step. In the lattice model, the ratio between chain and pair formation was determined at the M06-L level of theory. In the Mg system, the chain model has a binding energy of –69.4 kJ mol^–1^ while the pair model is –45.8 kJ mol^–1^. While the chain model showed a strong metal dependence in the binding energy, the pair model did not and the binding energy of nickel in the chain model is –46.4 kJ mol^–1^ per CO_2_, while the pair model has a binding energy of –47.2 kJ mol^–1^. If we use the nickel energetics as a basis for our lattice model, a Langmuir isotherm is observed (see Fig. 7 (bottom)). While this coarse grained model cannot tell us definitively what the mechanism for adsorption is in the Ni case, we clearly see that in order for a stepped isotherm to be observed, the energy to form a chain must be sufficiently stronger than forming pairs, short chains, or single site adsorption. This is consistent with recent experimental work done by Mason *et al.* in which the Long group proposes based on an infrared study that in mmen–Ni_2_(dobpdc), the carbamate group is on the terminal nitrogen.^9^

## Model and computational details

In this work we are interested in understanding the importance of the collective behavior of the amines on the adsorption isotherms for CO_2_. For this we developed a combined DFT lattice model approach, in which we used DFT calculation to estimate the different binding modes of CO_2_. These binding energies then serve as input for our lattice model.

### Model

Fig. 1 shows one channel of the mmen–M_2_(dobpdc) framework. All calculations were performed on the hexagonal unit cell containing six metal atoms and therefore six amine groups. The starting structure for the M_2_(dobpdc) frameworks was taken from experimental powder XRD data for the Mg system. Planas *et al.* had previously performed test calculations on cluster models of mmen–Mg_2_(dobpdc) with DFT using the PBE functional to determine the most favorable arrangement for mmen in Mg_2_(dobpdc).^13^ The amines were arranged in the periodic unit cell by hand in this most favorable arrangement as an initial guess prior to optimization.

### Density functional theory (DFT)

The M_2_–(dobpdc) MOF contains six unsaturated metal sites per unit cell. To calculate the binding energies of CO_2_ in its amine appended analogue mmen–M_2_(dobpdc), one mmen ligand per CO_2_ was added per unit cell. The smaller sized ethylenediamine (en) was used to saturate the remaining amines not involved in CO_2_ binding.

All DFT calculations were performed with periodic boundary conditions carried out using the VASP 5.3.3 package.^18,19^ The PBE and M06-L functionals^15,20,21^ were employed to examine the energetics of CO_2_ adsorption. On-site Hubbard U corrections were employed for metal d electrons. The U values are determined to reproduce oxidation energies in the respective metal oxides.^22^ This has been shown to lead to excellent binding energies for small molecules to open metal sites in M_2_-(dobdc) in prior work.^23^ The electron–ion interactions in these calculations were described with the projector augmented wave (PAW) method developed by Blöchl^24^ with an energy cutoff of 550 eV. The suitability of DFT (with PBE, PBE+U and M06-L among others) for studying gas adsorption in MOFs has been recently described.^25^ This combination of the PBE functional, PAW scheme, and energy cutoff was used for full geometry optimization of the various species investigated until the forces on all atoms were smaller than 0.05 eV Å^–1^. The sensitivity of binding energies to the sampling of the Brillouin-zone during geometry optimizations tested using the PBE functional at the *Γ*-point and while employing 1 × 1 × 3 and 2 × 2 × 2 Monkhorst–Pack *K*-point meshes. As the larger *K*-point meshes did not significantly change the reaction energies, only results obtained from *Γ*-point calculations are reported in the manuscript. Additional calculations were performed on the mmen–Mg_2_(dobpdc) framework with the PBE functional. First, a variety of configurations were explored to determine the CO_2_ binding. Likewise, infrared (IR) spectra were computed with density functional perturbation theory.^26^ Vibrational modes were computed for the amine, Mg and its first coordination sphere, as well as for bound CO_2_ when present. The remainder of the framework was kept rigid to reduce computational cost.

### Monte Carlo simulations

CO_2_ adsorption in mmen–M_2_(dobpdc) was studied by employing a lattice model. Each lattice point represents an amine fixed to a metal, and the energy of each site (its status) is determined by whether CO_2_ is bound at that point or not, the orientation of the amine with respect to the neighboring lattice sites, and whether CO_2_ is adsorbed or not on these neighboring sites. The energy contribution from each state was estimated based on the energy determined in the DFT calculations. The contribution in each state is given relative to the full-chain. We consider several different configurations: chain formation (amines with CO_2_ aligning in the *c*-direction), end-points (the start or end of a chain), pair-formation (two sites with CO_2_ interacting in either the *c*-direction or in the *ab*-plane), and, in addition, we have a small energy contribution for amines with CO_2_ bound but are not a part of a chain or a pair. It should be noted that the primary difference between the two pairing mechanisms is the distance between the two metals that anchor the amines. We expect this difference to result in changes on the order of a few kJ mol^–1^ and therefore is not significant enough to be represented in our coarse-grained model.

Adsorption isotherms are computed *via* grand-canonical Monte Carlo (GCMC) simulations, using the conventional acceptance rules.^17^ To make as direct a comparison of our lattice model with the experimental data as possible, we applied a shift of the pressure. This shift was obtained by fitting to the experimental isotherms for the highest and lowest temperatures. For the intermediate results we used a simple interpolation. The lattice model can in this way be related to models of the different metals and also test the limit of the phase-change behavior seen in experiments.

## Conclusions

New studies of CO_2_ adsorption on the amine-functionalized mmen–M_2_(dobpdc) framework have demonstrated that the previously proposed mechanism is not able to explain the metal dependence or the distinctive shape of the isotherm.^11^ As a result, our multi-scale study determined that a zwitterionic ammonium carbamate species formed by CO_2_ insertion in the M–N_amine_ bond is consistent not only with experimentally observed binding enthalpies but can also demonstrate that the resulting chain formation is responsible for the characteristic step in the isotherm. The chain model is further supported by the heat of adsorption, observed metal dependence, IR data, and the elongation of the M–N_amine_ bond distance upon CO_2_ adsorption, NEXAFS spectra, and ^15^N NMR data.^11^ Herein, we confirm that the long-range order imposed by the chain model is sufficient to cause the unusual step-shaped isotherm for mmen–M_2_(dobpdc). In contrast, the pair model produces a Langmuir-like isotherm. Furthermore, forming a long chain must be favorable over forming pairs. For all of the metals with the exception of Ni, the chain is favored and a stepped isotherm is observed. In mmen–Ni_2_(dobpdc), the binding energy in the chain is approximately the same as the energy to form a pair, resulting in favorable formation of pairs and a Langmuir like adsorption behavior.
