# Correction: CO_2_ induced phase transitions in diamine-appended metal–organic frameworks†
†Electronic supplementary information (ESI) available: VASP CONTCAR files and analogous CIF files are included for the optimized geometries for structures repeated as part of this correction. See DOI: 10.1039/c9sc90137j


**DOI:** 10.1039/c9sc90137j

**Published:** 2019-09-02

**Authors:** Bess Vlaisavljevich, Sondre K. Schnell, Allison L. Dzubak, Kyuho Lee, Nora Planas, Jeffrey B. Neaton, Laura Gagliardi, Berend Smit

**Affiliations:** a Department of Chemical and Biomolecular Engineering , University of California , 201 Gilman Hall , Berkeley , California 94720 , USA . Email: berend.smit@epfl.ch; b Department of Chemistry , Chemical Theory Center and Supercomputing Institute , University of Minnesota , Minneapolis , Minnesota 55455-0431 , USA . Email: gagliard@umn.edu; c Department of Chemistry , Norwegian University of Science and Technology , Høgskoleringen 5 , 7491 Trondheim , Norway; d Molecular Foundry , Lawrence Berkeley National Laboratory , One Cyclotron Road , Berkeley , California 94720 , USA; e Department of Physics , University of California , Berkeley , USA; f Kavli Energy NanoSciences Institute at Berkeley , Berkeley , CA , USA; g Institut des Sciences et Ingénierie Chimiques, Valais , Ecole Polytechnique Fédérale de Lausanne (EPFL) , Rue de l’Industrie 17 , CH-1950 Sion , Switzerland

## Abstract

Correction for ‘CO_2_ induced phase transitions in diamine-appended metal–organic frameworks’ by Bess Vlaisavljevich *et al.*, *Chem. Sci.*, 2015, **6**, 5177–5185.



## 

The authors regret that there are some discrepancies reproducing the data in the original article due to the determined coordinates not being the fully optimised geometries. The authors have provided more information as follows.

In the manuscript entitled ‘CO_2_ induced phase transitions in diamine-appended metal–organic frameworks’, minor errors with the attached coordinates and energies reported in the paper have recently been identified. In this communication, we correct these errors. Here, we present updated optimized geometries and binding energies. We also take this opportunity to include an extended computational details section to ensure reproducibility. In addition, we show that the overall conclusions of the paper are not affected by these changes.

A detailed comparison with the results reported by Lee *et al.*^1^ revealed that the DFT optimization of the coordinates provided with the manuscript do not lead to the values reported in the manuscript, and they warrant correction. Corrected coordinates and updated tables (Tables 1–7) and figures (Fig. 1, 2, 4 and 5) are included here for calculations using the PBE functional. These structures have been repeated using a slightly tighter force threshold than in the original manuscript (details below). The M06-L calculations reported in the original manuscript are not revisited since they were performed to assess the role of dispersion. Since the publication of our work in 2015, a far more detailed study of this effect has been published by one of the authors rendering these M06-L calculations unnecessary and we refer readers interested in the role of dispersion on the carbamate formation to this more recent study by Lee *et al.*^1^

**Table 1:** PBE binding energies including zero-point energy for the chain model plotted in Fig. 5 of the main text. In this data set, amines were attached to all six metal centers. There were five en-amines and one 1,1-dimethylenamine (mmen)

Metal	Δ*E* + ZPE (kJ mol^–1^)
Mg	68.0
Mn	63.3
Fe	57.2
Co	47.5
Ni	44.5
Zn	53.0

**Table 2:** Binding energies for the chain model with one mmen-amine and five en-amines per unit cell. The PBE functional was used

Metal	+*U* (eV)	Δ*E* (kJ mol^–1^)	ZPE (kJ mol^–1^)	Thermal correction (kJ mol^–1^)	Δ*E* + ZPE (kJ mol^–1^)	Δ*H* (kJ mol^–1^)
Mg	0.0	86.1	–8.9	3.8	77.2	81.1
Mn	3.8	73.8	–8.9	3.7	64.9	68.6
Fe	4.0	69.6	–8.2	3.7	61.4	65.1
Co	3.3	53.5	–7.8	3.5	45.7	49.2
Ni	6.4	48.3	–7.3	3.4	40.9	44.3
Zn	0.0	68.4	–8.5	3.6	59.9	63.5

**Table 3:** Binding energies for the chain model with only one mmen-amine per unit cell. No en-amines were included. The PBE functional was used

Metal	+*U* (eV)	Δ*E* (kJ mol^–1^)	ZPE (kJ mol^–1^)	Thermal correction (kJ mol^–1^)	Δ*E* + ZPE (kJ mol^–1^)	Δ*H* (kJ mol^–1^)
Mg	0.0	88.3	–8.8	3.7	79.5	83.1
Mn	3.8	78.4	–9.7	3.4	68.7	72.1
Fe	4.0	70.1	–7.7	3.4	62.3	65.7
Co	3.3	62.5	–7.4	3.3	55.1	58.4
Ni	6.4	51.0	–7.6	3.3	43.4	46.7
Zn	0.0	65.9	–8.4	3.3	57.5	60.8

**Table 4:** M–N_amine_ bond distances for the chain model. The PBE functional was used. Both the original and current values are reported in Angstroms

Metal	Orig. no CO_2_ (1 mmen/5 en)	Curr. no CO_2_ (1 mmen/5 en)	Curr. no CO_2_ (1 mmen/0 en)	Curr. chain (1 mmen/5 en)	Curr. chain (1 mmen/0 en)
Mg	2.44	2.460	2.402	4.433	4.298
Mn	2.44	2.460	2.402	4.405	4.364
Fe	2.39	2.461	2.393	4.458	4.397
Co	2.32	2.422	2.307	4.411	4.399
Ni	2.27	2.332	2.239	4.402	4.341
Zn	2.34	2.339	2.262	4.347	4.304

**Table 5:** PBE binding energies including zero-point energy for the chain model plotted in Fig. 5 of the main text. In this data set, amines were attached to all six metal centers. There were five en-amines and one 1,1-dimethylenamine (mmen)

Metal	Δ*E* + ZPE (kJ mol^–1^)
Mg	42.9
Mn	42.5
Fe	40.9
Co	41.5
Ni	41.3
Zn	39.9

**Table 6:** Binding energies per CO_2_ molecule for the pair model with only two mmen-amines per unit cell. No en-amines were included. The PBE functional was used

Metal	+*U* (eV)	Δ*E* (kJ mol^–1^)	ZPE (kJ mol^–1^)	Thermal correction (kJ mol^–1^)	Δ*E* + ZPE (kJ mol^–1^)	Δ*H* (kJ mol^–1^)
Mg	0.0	37.4	–9.5	3.6	28.0	31.5
Mn	3.8	40.2	–9.7	3.6	30.5	34.1
Fe	4.0	38.4	–9.7	3.6	28.7	32.2
Co	3.3	37.3	–9.7	3.6	27.6	31.2
Ni	6.4	36.6	–7.4	2.7	29.2	31.9
Zn	0.0	37.2	–9.5	3.5	27.6	31.2

**Table 7:** Binding energies for the first CO_2_ in the pair model with only two mmen-amines per unit cell. This can be thought of as single site adsorption or a ‘half-pair’. No en-amines were included. The PBE functional was used

Metal	+*U* (eV)	Δ*E* (kJ mol^–1^)	ZPE (kJ mol^–1^)	Thermal correction (kJ mol^–1^)	Δ*E* + ZPE (kJ mol^–1^)	Δ*H* (kJ mol^–1^)
Mg	0.0	51.4	–11.7	1.7	39.7	41.4
Mn	3.8	44.9	–12.2	2.0	32.7	34.7
Fe	4.0	50.1	–12.3	2.0	37.8	39.8
Co	3.3	52.4	–12.4	2.3	40.0	42.3
Ni	6.4	49.1	–11.5	1.8	37.6	39.4
Zn	0.0	48.3	–12.0	1.9	36.3	38.2

**Fig. 1:**
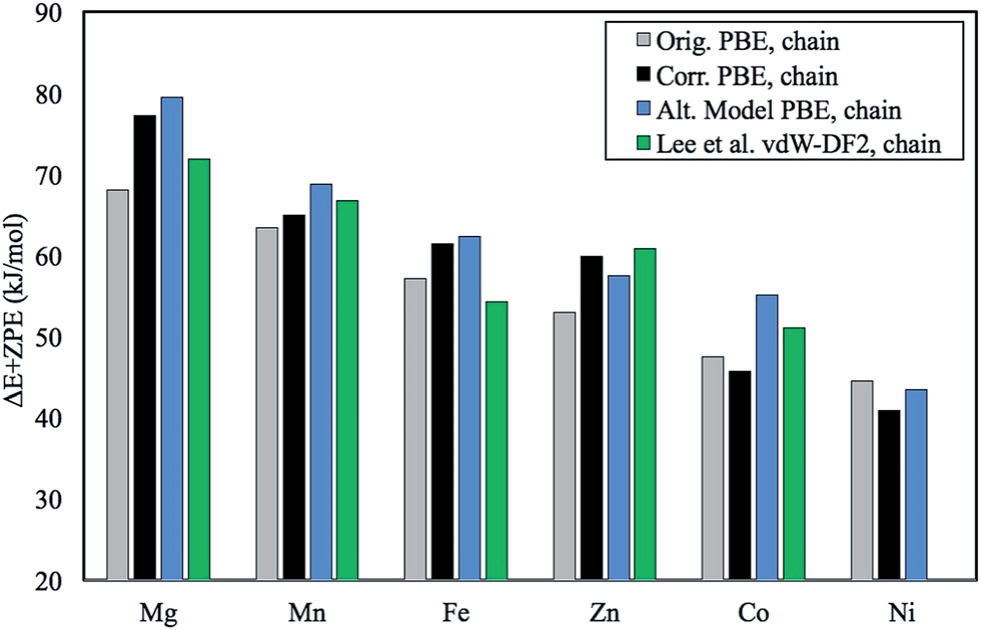
Plot of the original chain model Δ*E* + ZPE in kJ mol^–1^ in comparison with the corrected numbers for the same model. Also included is our previously unpublished “alternative chain model” and data from Lee *et al.*^1^ who employed the vdW-DF2 functional. Note that Lee *et al.* use 6 mmen-amines (1,1-dimethylenamine) per unit cell and the intermolecular interactions of amines across the *ab*-plane are treated more accurately due to a more extensive study with emphasis on understanding the role of these interactions.^1^ Additionally, Ni was not computed since it was shown to engage in single site adsorption and not chain formation shortly after the publication of the original work.^2^

**Fig. 2:**
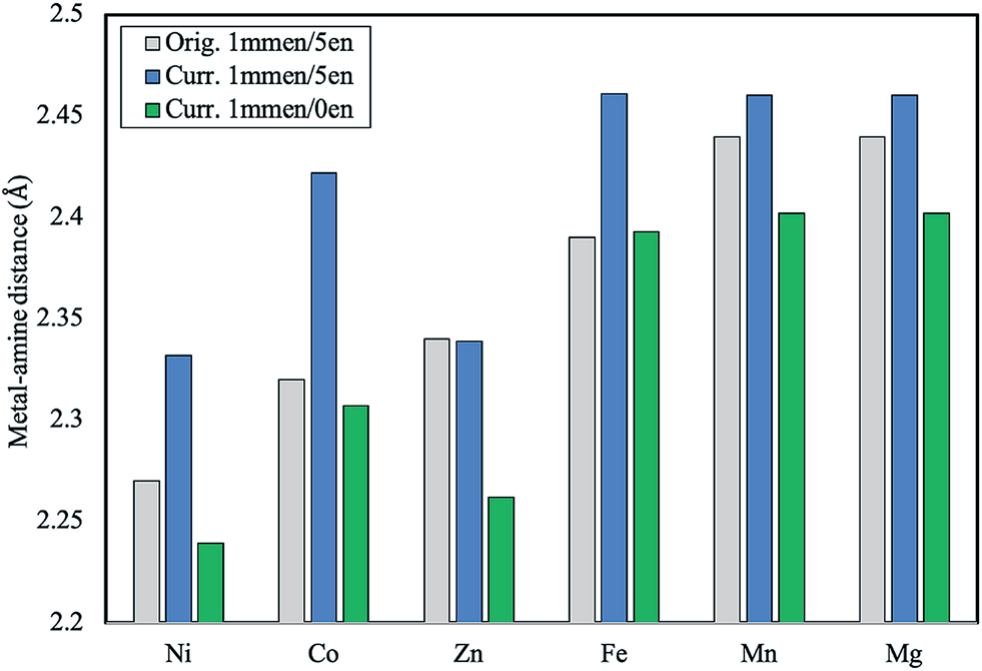
Plot of the M–N_amine_ distances in Å in the original model of mmen–M_2_(dobpdc) in comparison with the corrected numbers. Distances are reported for the structures prior to any CO_2_ being adsorbed.

**Fig. 3:**
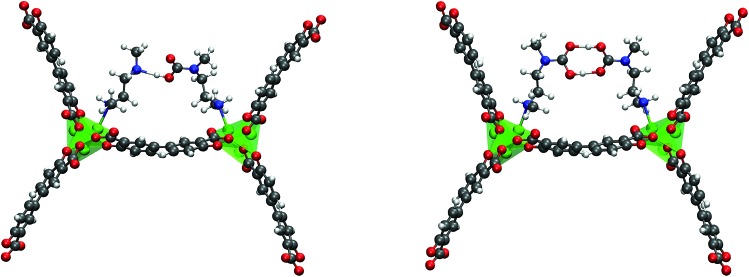
The organization of the carbamic acid groups in the pair model and the ‘half-pair’ single-site adsorption model. Corresponding energies are reported in Tables 5 and 7.

**Fig. 4:**
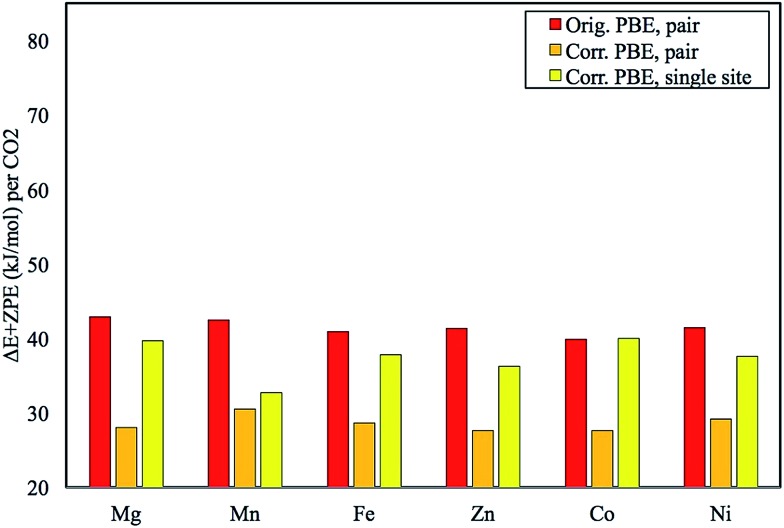
Plot of the original pair model Δ*E* + ZPE in kJ mol^–1^ in comparison with the corrected numbers. The results for the first step in pair formation, a single-site CO_2_ binding energy, are also given. Energies are reported per CO_2_ adsorbed.

**Fig. 5:**
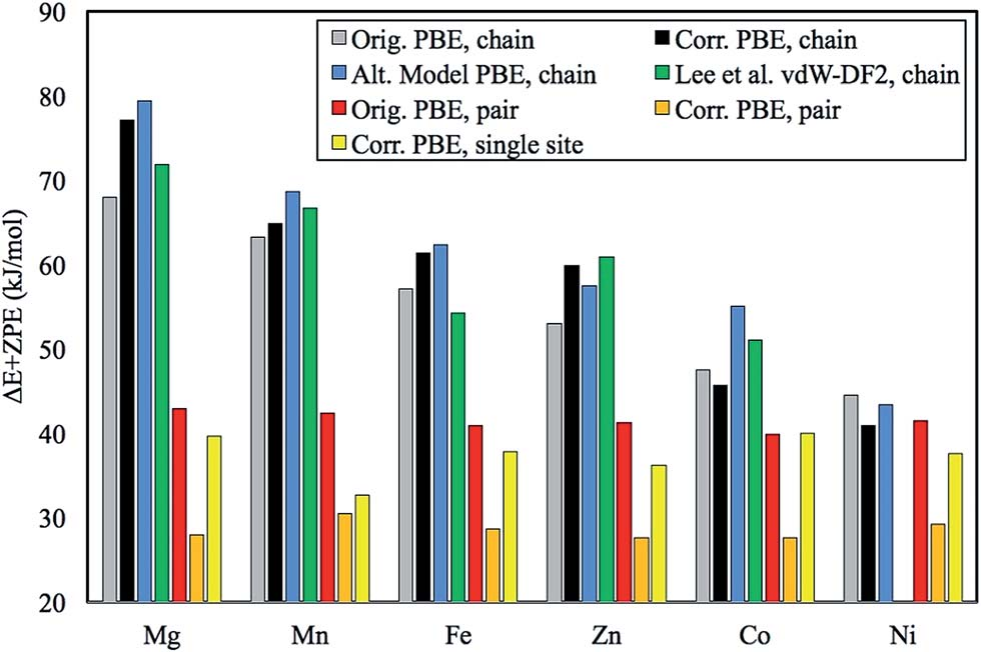
Plot of all of the original and corrected Δ*E* + ZPE in kJ mol^–1^ from Fig. 2 and 3.

In addition to correcting our DFT calculations, we examine the effects of the revised DFT values on the lattice model in this work. We recompute the lattice model with the M06-L and PBE values from the original manuscript as well as the corrected PBE values reported below (Fig. 6–8 and Tables 8–10). In all three sets of isotherm plots the ordering is preserved but the inflection points are spaced differently with the new PBE numbers, leading to quantitative differences that are nonetheless qualitatively similar to previous work.

**Fig. 6:**
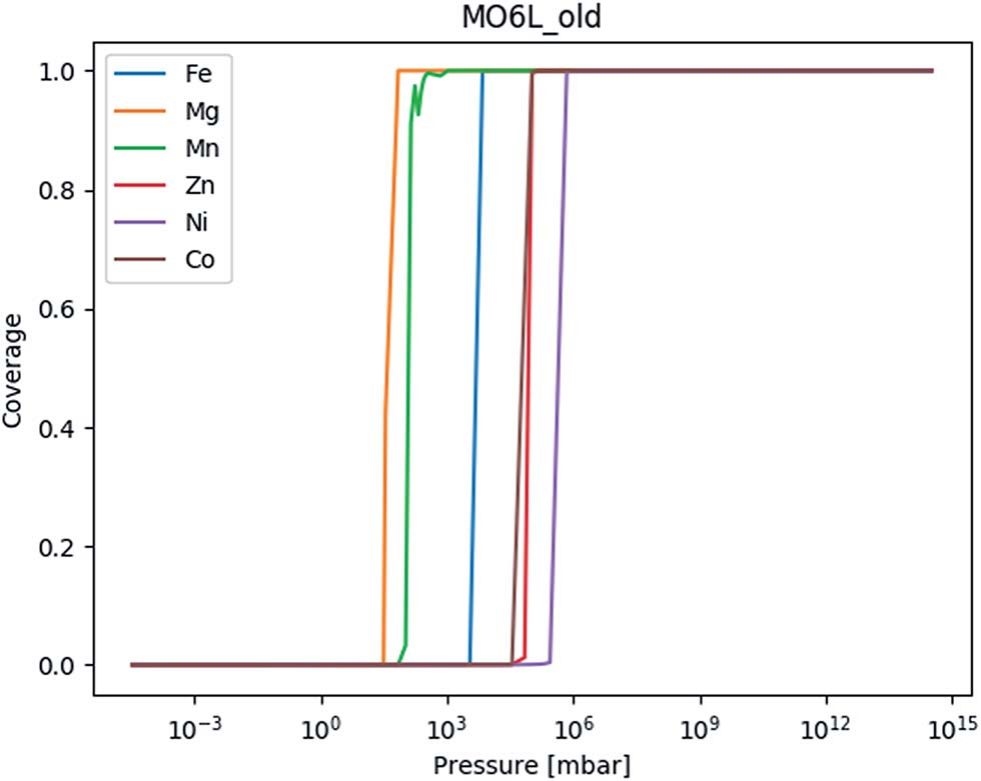
The adsorption isotherm based on the chain for mmen–Mg_2_(dobpdc) based on the M06-L values in the original manuscript.

**Fig. 7:**
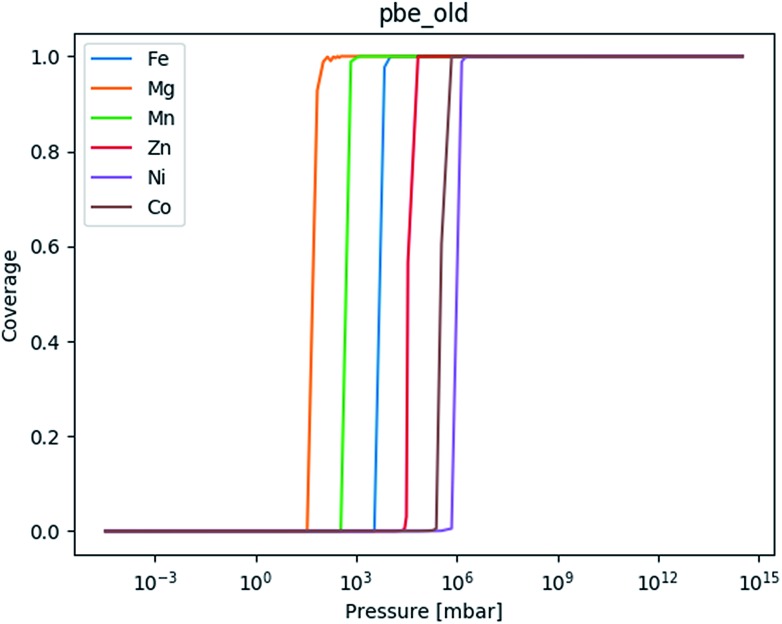
The adsorption isotherm based on the chain for mmen–Mg_2_(dobpdc) based on the PBE values in the original manuscript.

**Fig. 8:**
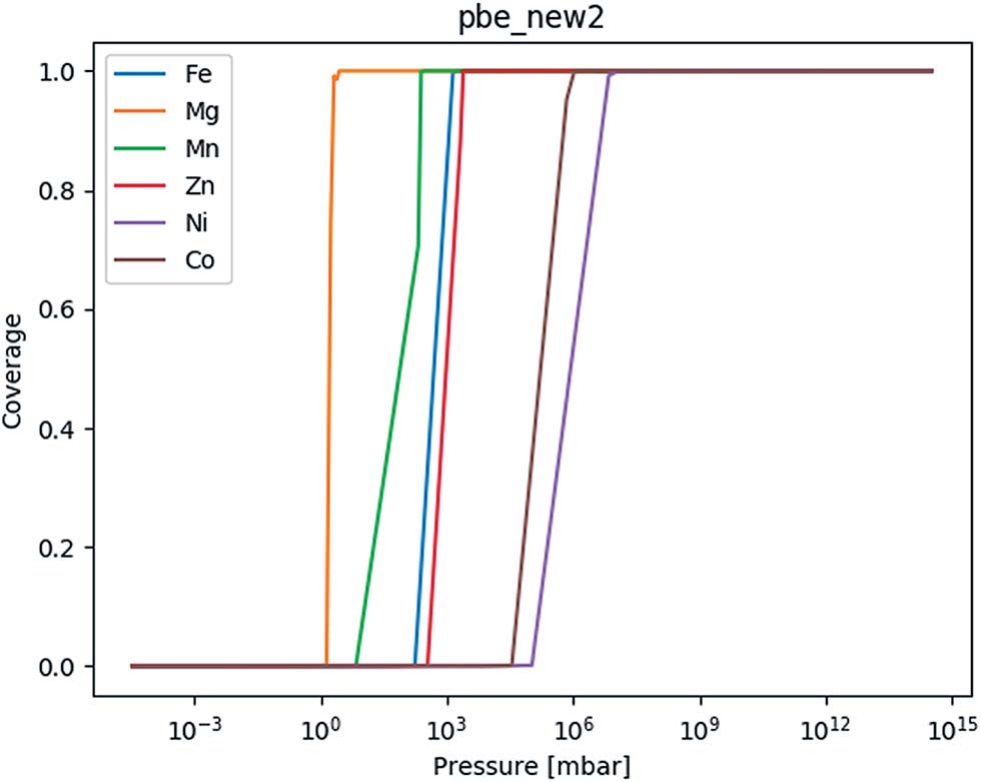
The adsorption isotherm based on the chain for mmen–Mg_2_(dobpdc) based on the PBE values obtained as part of this erratum (Table 5).

**Table 8:** Energy contributions to the coarse-grained lattice model in Fig. 6. Energy contributions based on calculations using the M06-L functional from the original work

Amine site	Neighboring amine	Mg	Mn	Fe	Co	Ni	Zn
Free amine, no CO_2_	N/A	0	0	0	0	0	0
End-point of a chain	N/A	80	77	66	58	54	58
Isolated amine	N/A	24	24	24	24	24	24
Middle of a chain	N/A	100	96	83	73	67	73
Pair *c*-direction (CO_2_)	Pair *c*-direction (CO_2_)	66	60	62	67	68	61
Pair *c*-direction (no CO_2_)	Pair *c*-direction (CO_2_)	33	30	31	33	33	30
Pair *c*-direction (CO_2_)	Pair *c*-direction (no CO_2_)	33	30	31	33	33	30
Pair *c*-direction (no CO_2_)	Pair *c*-direction (no CO_2_)	0	0	0	0	0	0
Pair *ab*-plane (CO_2_)	Pair *ab*-plane (CO_2_)	66	60	62	67	68	61
Pair *ab*-plane (no CO_2_)	Pair *ab*-plane (CO_2_)	33	30	31	33	33	30
Pair *ab*-plane (CO_2_)	Pair *ab*-plane (no CO_2_)	33	30	31	33	33	30
Pair *ab*-plane (no CO_2_)	Pair *ab*-plane (no CO_2_)	0	0	0	0	0	0

**Table 9:** Energy contributions to the coarse-grained lattice model in Fig. 6. Energy contributions based on calculations using the PBE functional from the original work

Amine site	Neighboring amine	Mg	Mn	Fe	Co	Ni	Zn
Free amine, no CO_2_	N/A	0	0	0	0	0	0
End-point of a chain	N/A	78	73	66	54	51	61
Isolated amine	N/A	24	24	24	24	24	24
Middle of a chain	N/A	98	91	82	68	64	76
Pair *c*-direction (CO_2_)	Pair *c*-direction (CO_2_)	62	61	59	69	69	57
Pair *c*-direction (no CO_2_)	Pair *c*-direction (CO_2_)	31	30	29	35	35	29
Pair *c*-direction (CO_2_)	Pair *c*-direction (no CO_2_)	31	30	29	35	35	29
Pair *c*-direction (no CO_2_)	Pair *c*-direction (no CO_2_)	0	0	0	0	0	0
Pair *ab*-plane (CO_2_)	Pair *ab*-plane (CO_2_)	62	61	59	69	69	57
Pair *ab*-plane (no CO_2_)	Pair *ab*-plane (CO_2_)	31	30	29	35	35	29
Pair *ab*-plane (CO_2_)	Pair *ab*-plane (no CO_2_)	31	30	29	35	35	29
Pair *ab*-plane (no CO_2_)	Pair *ab*-plane (no CO_2_)	0	0	0	0	0	0

**Table 10:** Energy contributions to the coarse-grained lattice model in Fig. 6. Energy contributions based on calculations using the PBE functional from this correction

Amine site	Neighboring amine	Mg	Mn	Fe	Co	Ni	Zn
Free amine, no CO_2_	N/A	0	0	0	0	0	0
End-point of a chain	N/A	89	75	70	53	47	69
Isolated amine	N/A	24	24	24	24	24	24
Middle of a chain	N/A	111	94	88	66	59	86
Pair *c*-direction (CO_2_)	Pair *c*-direction (CO_2_)	80	88	82	40	84	80
Pair *c*-direction (no CO_2_)	Pair *c*-direction (CO_2_)	40	44	41	40	42	40
Pair *c*-direction (CO_2_)	Pair *c*-direction (no CO_2_)	40	44	41	40	42	40
Pair *c*-direction (no CO_2_)	Pair *c*-direction (no CO_2_)	0	0	0	0	0	0
Pair *ab*-plane (CO_2_)	Pair *ab*-plane (CO_2_)	80	88	82	40	84	80
Pair *ab*-plane (no CO_2_)	Pair *ab*-plane (CO_2_)	40	44	41	40	42	40
Pair *ab*-plane (CO_2_)	Pair *ab*-plane (no CO_2_)	40	44	41	40	42	40
Pair *ab*-plane (no CO_2_)	Pair *ab*-plane (no CO_2_)	0	0	0	0	0	0

Finally, we discuss different ways that CO_2_ can coordinate to the metal binding site, as shown in Fig. 3. We should have noted more clearly in our manuscript that these were starting configurations and not necessarily the final converged structures since our goal was to try several starting geometries to determine which coordination environment around the metal site was lowest in energy. Take for example bidentate insertion. Chemical intuition suggests that this structure could rotate to one that has only one CO_2_ oxygen center closer to the metal than the other and we observe this in our optimized structure. The resulting geometries we obtained for the starting arrangements noted in the figure are higher in energy than the chain model as reported in our original paper. We wish to emphasize that at the time of our 2015 study, our objective was to understand whether or not CO_2_ was bound to the metal and if one-dimensional chain formation could lead to a step in the adsorption isotherm. It has since become clear that a far more thorough study of the arrangements of the amines is required to truly understand competing amine arrangements preset in experiment. This was outside the scope of our work. Once more, these calculations are perhaps now outdated given work in the field in recent years. We again refer interested readers to a more recent study by Lee *et al.*^1^

## 1. Extended computational details to ensure reproducibility

In the course of rectifying the error in our calculations, we wanted to ensure that all revised calculations were converged using the exact same protocol; therefore, we repeated the PBE calculations for the pair and chain models using updated computational details given here to ensure reproducibility.

The M_2_(dobpdc) MOF contains six unsaturated metal sites per unit cell. To calculate the binding energies of CO_2_ in its amine appended analogue mmen–M_2_(dobpdc), one mmen ligand per CO_2_ was added per unit cell. The smaller sized ethylenediamine (en) was used to saturate the remaining amines not involved in CO_2_ binding. In the case of the pair mode, two mmen-amines are included per unit cell only. All DFT calculations were performed with periodic boundary conditions carried out using the VASP 5.4.4 package (original calculations were performed with VASP 5.3.3). The PBE functional was employed to examine the energetics of CO_2_ adsorption.^3^ On-site Hubbard *U* corrections were employed for metal d electrons.^4^ The *U* values are determined to reproduce oxidation energies in the respective metal oxides and are given in the tables below. The electron–ion interactions in these calculations were described with the projector augmented wave (PAW) method developed by Blöchl with an energy cutoff of 550 eV.^5^ This combination of the PBE functional, PAW scheme, and energy cutoff was used for full geometry optimization of the various species investigated until the forces on all atoms were smaller than 0.02 eV Å^–1^ and the SCF convergence was set to 1 × 10^–7^ eV. Given the large size of the unit cell and the tests with other numbers of *K*-points from the original study, only results obtained from *Γ*-point calculations are reported here.

Finally, heats of adsorption are now reported below along with *E* + ZPE values, while in the original manuscript only *E* + ZPE were reported. No changes were made to how the vibrational corrections were computed; however, we have included some additional details to ensure reproducibility.^6^ Harmonic vibrational modes (*ω*_*i*_) were computed for CO_2_ in the gas phase and its bound product state (amine–CO_2_–MOF complex). The framework itself was taken to be rigid and only the vibrational modes associated with the motion of the amine, the metal center, first coordination sphere (oxygen atoms bound to the metal in the MOF backbone), and (if present) the bound CO_2_ were computed. Since the harmonic approximation breaks down for low frequency modes, we replaced all modes less than 50 cm^–1^ with 50 cm^–1^ when computing the zero-point and thermal energies. The following standard harmonic expressions were used to compute the vibrational corrections:

Zero-point vibrational energy (ZPE) is:
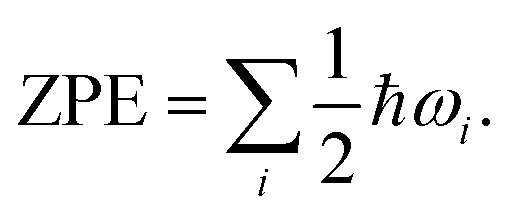


For CO_2_ in the gas phase, the thermal correction to the energy was taken to be:
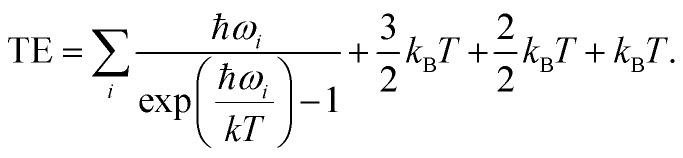


While for the bound product, the rotational and translational degrees of freedom of CO_2_ have been converted to additional vibrational modes allowing one to compute the thermal correction simply as:
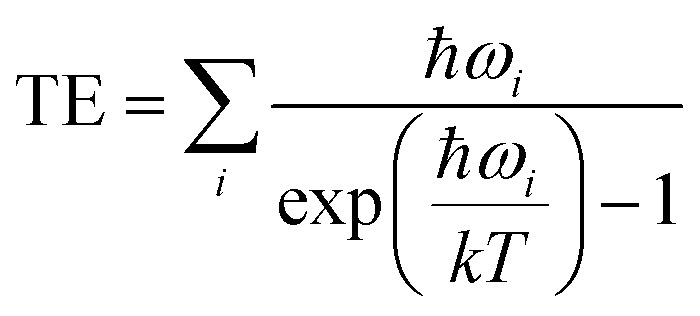


## 2. Values for the chain model

The chain model used in our original study included 1 mmen- and 5 en-amines. The values from the original paper are reported in Table 1.

When we repeat these calculations using the procedure described in Section 1, we obtain the values in Table 2.

In addition to the chain model described above (1 mmen- and 5 en-amines per unit cell), during our original study we performed calculations with another model that was not included in the manuscript since its values yielded results further from experiment. This model includes only 1 mmen-amine per unit cell (no other amines) and was used to test the assumption that the five en-amines are indeed spectators with respect to the metal dependence of the binding energy. We present the results from this model in Table 3.

In the original paper we noted that the energy and bond length trends are correlated and are consistent with the Irving–Williams series. This is no longer true for all metals under investigation, with Zn being an outlier. The results for Zn can be explained by more recent work.^1^

## 3. Values for the pair model

The model used to compute the “pair” adsorption mechanisms included 2 mmen-amines and 0 en-amines. The values in the original paper are presented in Table 5.

The calculations were repeated for the pair model and are presented in Table 6.

As part of our initial study, we included a lattice model. This model included pair interactions where only one of the amines involved in the pair has a CO_2_ bound. We did not include the DFT values from these calculations in the original manuscript but include them here for completeness.

## 4. Lattice model plots

The lattice models to generate adsorption isotherms for these systems were run at one temperature (∼25 °C) using four different input parameters. First the M06-L and PBE values from the original paper were used once more as it has been some time since we have run the lattice model. Then the model is repeated with the new set of values from PBE.

If we compare Fig. 7 and 8, the order is preserved, but the infliction points are spaced a bit differently. This is due to the scaling factor being constant and is something we scaled for each of the different systems as well. The slope is also a bit different, but not more then we should expect for this simple lattice model. Furthermore, we only ever aimed to reproduce the step and the order of the metals. Any finer details cannot be expected to be obtained from this model. The exact values used to compute the isotherms are given in the tables below.

The Royal Society of Chemistry apologises for these errors and any consequent inconvenience to authors and readers.

## Supplementary Material

Supplementary information
