# Supplementary material for: CO2 induced phase transitions in diamine-appended metal–organic frameworks
Source: Chem Sci. 2015 Jun 17;6(9):5177–85. doi: 10.1039/c5sc01828e (PMC5500831; doi:10.1039/c5sc01828e)
Supplement: Supplementary file 2 [file SC-006-C5SC01828E-s002.pdf]

Electronic Supplementary Material (ESI) for Chemical Science.
This journal is © The Royal Society of Chemistry 2015

Supporting information for: CO_2 induced phase transitions in diame-appended metal organic frameworks

Bess Vlaisavljevich[1,‡], Samuel O. Odoh[2,‡], Sondre Schnell,[1,3] Allison L. Dzubak,[2] Nora Planas,[2] Kyuho Lee[4], Jeff Neaton[4], Berend Smit[1,5,*] and Laura Gagliardi[1,*]

1. Department of Chemical and Biomolecular Engineering, University of California, 201 Gilman Hall, Berkeley, California, 94720, United States. 2. Department of Chemistry, Chemical Theory Center, and Supercomputing Institute, University of Minnesota, Minneapolis, Minnesota 55455-0431, United States. 3. Department of Chemistry, Norwegian University of Science and Technology, Høgskoleringen 5, 7491 Trondheim, Norway. 4. Molecular Foundry, Lawrence Berkeley National Laboratory, One Cyclotron Road, Berkeley, California, 94720, United States. 5. Institut des Sciences et Ingénierie Chimiques, Valais, Ecole Polytechnique Fédérale de Lausanne (EPFL), Rue de lIndustrie 17, CH-1950 Sion, Switzerland.

E-mail: berend-smit@berkeley.edu,gagliard@umn.edu

*To whom correspondence should be addressed

Table S1: Values for the calculated and experimental adsorption energies in kJ/mol plotted in Figure 5.

	Pair Model		Chain Model	
Metal	PBE	M06L	PBE	M06L
Mg	42.9	45.8	68.0	69.4
Mn	42.5	41.2	63.3	66.8
Fe	40.9	43.1	57.2	57.7
Co	41.5	46.5	47.5	50.8
Ni	41.3	47.2	44.5	46.4
Zn	39.9	42.6	53.0	50.8

Table S2: Values for the calculated and experimental M-N_{amine} bond distances from mmen-M_2(dobpdc) in Å plotted in Figure 5.

Metal	PBE	M06L
Mg	2.44	2.39
Mn	2.42	2.36
Fe	2.39	2.34
Co	2.32	2.28
Ni	2.27	2.25
Zn	2.34	2.30

Table S3: Energy contributions to the coarse-grained lattice model.

Type of Interaction		Percentage of Energy					
Amine Site	Neighboring Amine	Mg	Mn	Fe	Co	Ni	Zn
Free amine, no CO_2	N/A	0	0	0	0	0	0
End-point of a chain	N/A	80	80	80	80	80	80
Isolated amine	N/A	24	24	24	24	24	24
Middle of a chain	N/A	100	96	83	73	67	73
Pair C-direction (CO_2)	Pair C-direction (CO_2)	66	60	62	67	67	61
Pair C-direction (No CO_2)	Pair C-direction (CO_2)	33	30	31	33	33	30
Pair C-direction (CO_2)	Pair C-direction (No CO_2)	33	30	31	33	33	30
Pair C-direction (No CO_2)	Pair C-direction (No CO_2)	0	0	0	0	0	0
Pair ab-plane (CO_2)	Pair ab-plane (CO_2)	66	60	62	67	67	61
Pair ab-plane (No CO_2)	Pair ab-plane (CO_2)	33	30	31	33	33	30
Pair ab-plane (CO_2)	Pair ab-plane (No CO_2)	33	30	31	33	33	30
Pair ab-plane (No CO_2)	Pair ab-plane (No CO_2)	0	0	0	0	0	0

The 80% contribution for end point energies was a guess based on the behavior of the model and estimations based on large DFT calculations (we expanded the unit cell in the c-direction and considered the case where every other amine had CO_2 bound).

Based on this DFT calculation, we expect that the reduction in energy should be between 60-90% of the full chain. However, if the end point energy is set too low (<70%), the step in the adsorption behavior becomes extremely abrupt, as it becomes very favorable for the system to form as long a chain as possible. This can be seen as steps, since the system is a full chain and will not adsorb more until it can form a full new chain. If it is set above 90%, the system approaches Langmuir-like behavior, since there is no reason to terminate the chain at all. We think this site makes a good contribution to forming a full chain, since it has a CO_2 fully adsorbed. At the same time it should not be as good as a molecule in a fully formed chain. The energy of an isolated CO_2 was based on experimental measurements of the isosteric heat of adsorption at very low loading. This value (17 kJ/mol) was taken from Figure 3 in McDonald *et al. J. Am. Chem. Soc. 2012, 134, 7056-7065.*
